# A PCR assay detects a male-specific duplicated copy of Anti-Müllerian hormone (*amh*) in the lingcod (*Ophiodon elongatus*)

**DOI:** 10.1186/s13104-016-2030-6

**Published:** 2016-04-22

**Authors:** Eric B. Rondeau, Cassandra V. Laurie, Stewart C. Johnson, Ben F. Koop

**Affiliations:** Department of Biology, Centre for Biomedical Research, University of Victoria, Victoria, BC V8W 3N5 Canada; Pacific Biological Station, Fisheries and Oceans Canada, Nanaimo, BC V9T 6N7 Canada

**Keywords:** Sex determination, *amh*, Anti-Müllerian hormone, Müllerian inhibiting substance, *mis*, Lingcod, *Ophiodon elongatus*

## Abstract

**Background:**

Anti-Müllerian hormone (*amh*) or Müllerian-inhibiting substance (*mis*) is a member of the transforming growth factor-β family of hormones. This gene plays a key role in vertebrate male sex-determination by inhibiting the development of the Müllerian ducts, and has been shown to be the master sex-determinant in the Patagonian pejerrey.

**Results:**

In the lingcod, *Ophiodon elongatus*, both males and females share one copy of *amh*, however we have identified a second duplicate copy that appears solely in the male individuals. We have developed a PCR-based assay targeting the TGF-β domain of *amh* that provides a simple method with which to sex lingcod from a small amount of tissue. An analysis across 57 individuals gave a 100 % success rate in identifying the phenotypic sex.

**Conclusions:**

We present a simple method to sex lingcod through non-lethal tissue sampling. A third, independent, male-specific duplication of *amh* in a teleost fish has been identified in the lingcod.

**Electronic supplementary material:**

The online version of this article (doi:10.1186/s13104-016-2030-6) contains supplementary material, which is available to authorized users.

## Findings

Teleosts do not share a common master sex-determination gene, although certain gene families are consistently implicated for this role. The transforming growth factor-β family contributes two confirmed Master sex determinants (MSD), Gonadal soma-derived factor [1] and Anti-müllerian hormone (*amh*) (reviewed in [2]). Male-specific duplicated copies of *amh* (also known as Müllerian-inhibiting substance) have been implicated in controlling sex-determination in the Patagonian pejerrey *Odontesthes hatcheri* [3] and *amh* is essential for male sex-determination in the Nile tilapia *Oreochromis niloticus* [4, 5]. In this work, we show that a male-specific duplicated copy of *amh* is also strongly linked to sex in the lingcod (*Ophiodon elongatus*).

The lingcod is a gonochorisitic, bottom-dwelling member of the greenling family (Hexagrammidae) that is found in nearshore marine areas along the west coast of North America [6]. Lingcod form an important component of the commercial, recreational and aboriginal (CRA) groundfish fisheries, with commercial lingcod fisheries forming the ninth most valuable commercial fishery in Pacific Canada in 2013 [7]. This fishery is considered to be sustainably managed yet certain stocks have still become depressed, primarily along the inside passage of Vancouver Island [8]. Given the uncertain nature of the inside stocks and the suggestion of asymmetric migration [9], genetic tools are an important resource that can aid in more comprehensive stock assessment and management.

As mature males have a distinct papilla just forward of the anus it is possible to assign sex of mature lingcod based on size and the presence or absence of this external feature. However, to determine the sex of immature individuals and to confirm the sex of mature lingcod requires euthanization and examination of the gonads. The development of this non-lethal method for the assignment of sex is a valuable tool to support ecological and stock assessment studies of lingcod where examination of the gonads for sex is not possible or desirable.

Samples were collected in 2013 and 2014 by the Government of Canada as part of stock assessment activities; sampling was exempt from requiring an animal use protocol under section 4.1.2.2 of the Canadian Council on Animal Care (CCAC), as determined by Fisheries and Oceans Canada. Sample collection data is summarized in Additional file 1. Genomic DNA was extracted from ethanol-preserved fin clips using DNeasy kits (Qiagen). Samples were sexed by dissection and examination of the gonads by qualified groundfish biologists and technicians.

**Fig. 1:**
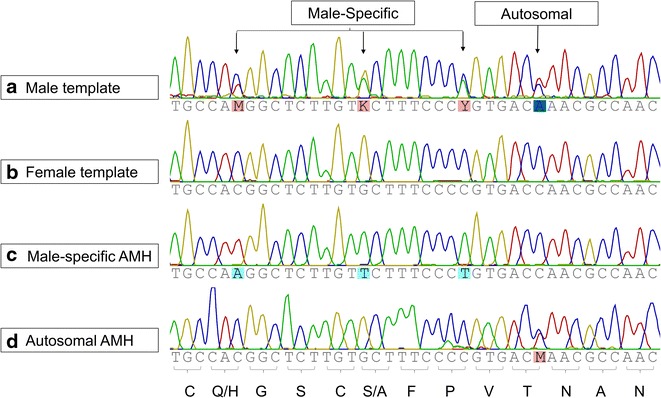
An aligned portion of the TGF-β domain. Primers that amplify both copies of *amh* were used to amplify **a** a male template and **b** a female template. **c** Male-specific *amh* primers and **d** Autosomal *amh* primers were used on a male template. Male-specific and autosomal variable sites are noted with *arrows* and the translated consensus is shown along the * bottom*. Image produced using Geneious v8.0.4 (http://www.geneious.com [12]), sequences are from 1873–1912 bp in KP686073 and 1871–1910 bp in KP686074

Initial PCR amplifications were conducted and sequenced using primers designed to conserved portions of sablefish (GenBank:GAJJ01025678 [10] and stickleback (Ensembl:ENSGACT00000016731; ensembl.org) *amh* transcripts (primers and methods in Additional file 2). Male *amh* sequences appeared to be highly polymorphic (Fig. 1a), while female *amh* sequences appeared to be highly conserved (Fig. 1b), which suggested that these primers may amplify two loci in males. The sequences were extended from the original PCRs using genome walking methods, with primers and protocol in Additional file 2 [11]. These primers were used to isolate male-specific (GenBank:KP686073) and shared (GenBank:KP686074) partial *amh* gene sequences (Additional file 3). Of the seven exons expected based on sablefish and threespine stickleback, the sequences have been generated from the middle of exon 3 through exon 7 and into the 3′-untraslanted region (UTR). Genome walking consistently failed in the intron between exons 2 and 3 due to repetitive sequence, leaving the partial sequences presented.

From these extended sequences, primers were then designed to target either the male-specific (Fig. 1c) or the shared copy of *amh* (Fig. 1d) from the male individual used in Fig. 1a. Based on the detection of two alleles (i.e., two copies) at the autosomal polymorphic site (Fig. 1d), it is suggested that the male-specific copy is not a third allele, but a duplicated copy that appears solely on an emerging Y-chromosome. Further evidence is given by the significantly different 3′UTRs in the sequences generated by genome walking (see alignment in Additional file 3). Analysis of the TGF-β domain shows that while coding changes exist (9/93 sites), the seven conserved cysteines (Prosite PDOC00223) required for inter and intra-chain disulfide bonds are conserved in both sequences (Additional file 4). Unlike in tilapia [4], it appears that the TGF-β domain of the male-specific duplicate has remained functional in the lingcod.

**Fig. 2:**
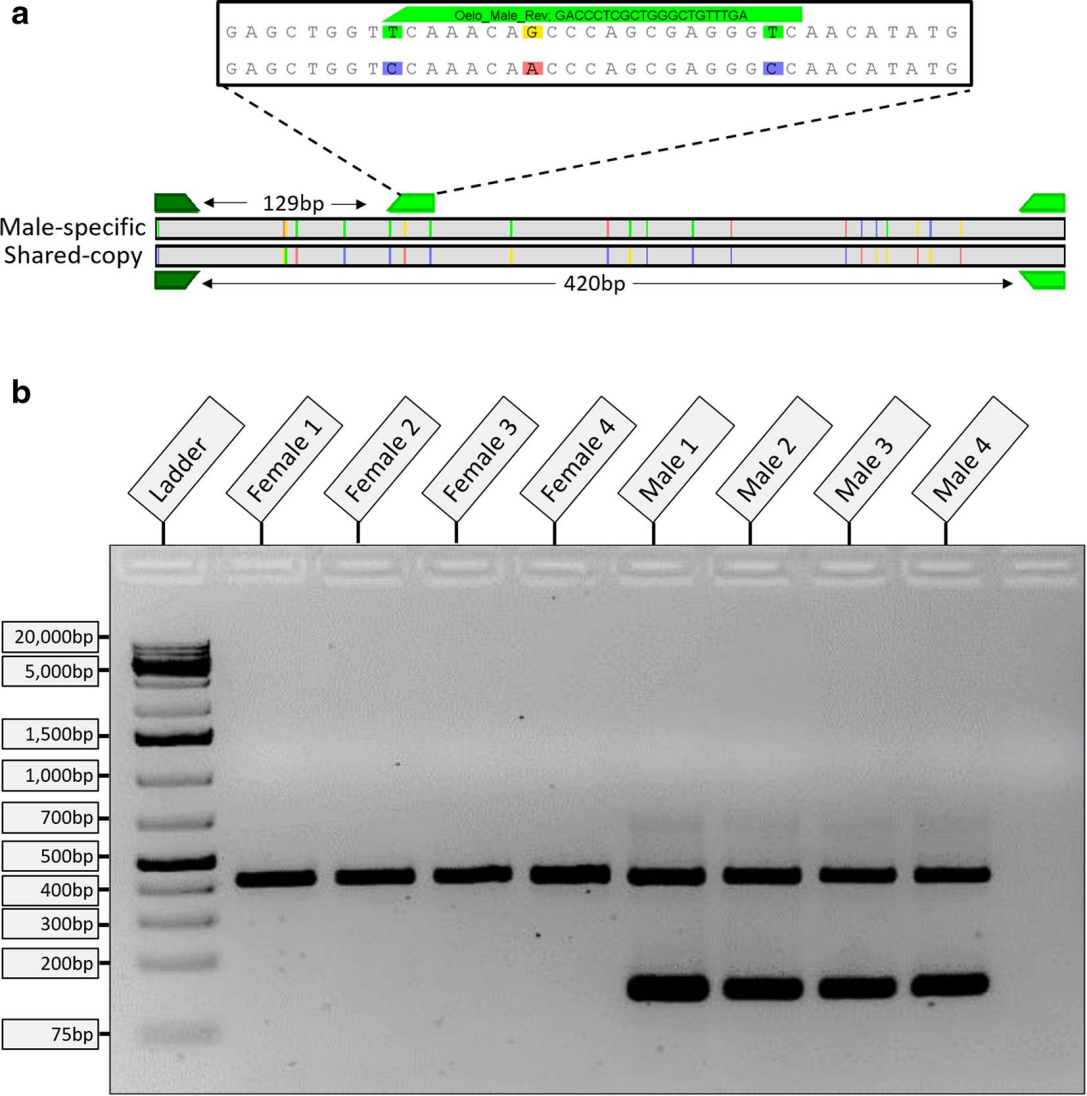
Testing the lingcod PCR assay. The PCR assay strategy is shown in **a**. The external primers (*green polygons*) bind both versions while the internal primer binds only in the male due to three internal mismatches. Mismatches are *coloured lines*, image produced in Geneious v8.0.4 [12]. In **b**, amplification of four male and four female lingcod templates using the Oelo-Sex three primer PCR assay. Presence of the 129 bp band denotes the presence of the male-specific copy of *amh*, while the 420 bp band is the shared autosomal copy. Ladder used is 1 kb O’generuler plus (Thermo), 2.0 % agarose gel stained with ethidium bromide

We have developed a PCR assay to sex individual lingcod on an agarose gel. Oelo_Sex_For (5′-TYTAGAACAGGGGAGAGCCA-3′) and Oelo_Sex_Rev (5′-TTAGTTTTAGCGGCATCCAC-3′) amplifies both *amh* copies as a 420 bp band, while Oelo_Male_Rev (5′-GACCCTCGCTGGGCTGTTTGA-3′) with Oelo_Sex_For amplifies only the male version, as a 129 bp band. The 10 μl reactions containing 1X Hot Start PCR Buffer, 0.25 μM of each of the two reverse primers (IDT), 0.5 μM of the forward primer (IDT), 2.0 mM MgCl_2_, 0.2 mM dNTPs, and 0.5U Maxima Hot Start Taq (Thermo) were incubated for 4 min at 95 °C, then 35 cycles of 30 s at 95 °C, 30 s at 58 °C, 30 s at 72 °C, and a final extension of 10 min at 72 °C. Amplified PCR fragments were then run on a 2.0 % agarose/1XTAE gel stained with EtBr at 100 V for 45 min along with 2.5 μl O’GeneRuler™ 1 kb Plus DNA Ladder (Thermo). These reactions successfully amplified a single band in females and two bands in males (Fig. 2). We positively identified phenotypic sex in 57 individuals (29 males, 28 females) using this PCR and gel based assay. A further seven unsexed museum specimens (Additional file 1) were screened and the male-specific sequence was found in samples from both Washington State and Alaska, confirming the distribution of this sequence across a large geographic range.

Here, we provide a simple, genetic method to sex lingcod through non-lethal tissue sampling. Although the role of *amh* as a MSD in lingcod remains to be confirmed, our work shows that a third, independent, male-specific duplication of *amh* in a teleost fish has occurred, suggesting that multiple evolutions of the *amh* gene as a novel MSD may have occurred in multiple teleost lineages.

## Availability of supporting data

The data supporting the results of this article are included within the article and its additional files, or uploaded to NCBI, accessions GenBank: KP686073-KP686074.

## Additional files

10.1186/s13104-016-2030-6 Sampling information. Additional information on the collection of lingcod tissues used in the study.
10.1186/s13104-016-2030-6 Primers and protocol. Primers and protocol used to amplify lingcod *amh* KP686073 and KP686074.
10.1186/s13104-016-2030-6 Partial *amh* gene sequences. Sequences of male-specific (KP686073) and shared (KP686074) partial *amh* gene sequences and corresponding alignment (Geneious v8.1.7 default alignment settings).
10.1186/s13104-016-2030-6 TGF-β domain. Translation of TGF-β domain and identification of conserved cysteines between *amh* and *amhy*.

## Abbreviations

*Amh*: Anti-Müllerian hormone
*Mis*: Müllerian inhibiting substance
MSD: master-sex determinant
TGF-β: transforming growth factor beta
UTR: untranslated region
