# Supplementary material for: A PCR assay detects a male-specific duplicated copy of Anti-Müllerian hormone (amh) in the lingcod (Ophiodon elongatus)
Source: BMC Res Notes. 2016 Apr 22;9:230. doi: 10.1186/s13104-016-2030-6 (PMC4840878; doi:10.1186/s13104-016-2030-6)
Supplement: Supplementary file 2 — 10.1186/s13104-016-2030-6 Primers and protocol. Primers and protocol used to amplify lingcod amh KP686073 and KP686074. [file 13104_2016_2030_MOESM2_ESM.doc]

Additional file 2: Primers and protocol used to amplify lingcod AMH KP686073 and KP686074

1. Initial amplifications of Lingcod AMH were performed using AMH_F3 (5’-CTGCTGAAGGCCCTGCAGACG-3’) and AMH_R1 (5’-TACGCCACCGGCACGCAGCA-3’), originally designed for Sablefish (*Anoplopoma fimbria*). The PCR reaction was performed with 1X Hot Start PCR Buffer, 0.5 µM of forward and reverse primers, 2.0 mM MgCl_2_, 0.2 mM dNTPs, and 0.5 units Maxima Hot Start Taq DNA Polymerase as 10 μl reactions with approximately 30 ng of DNA. PCR amplification on all lingcod templates with AMH F3 and AMH R1 on a Techne TC-412 thermocycler at 95°C for 4 minutes then 35 cycles of 95°C for 30 seconds, 55°C annealing for 45 seconds, 72°C 1 minute 30 second extension, then 72°C for 10 minutes, and kept at 4 °C. Clean up was performed as using Exonuclease I and Fast Alkaline phosphatase (Thermo); 5 µl of each PCR product, 0.5 µl of exonuclease I and 1.0 µl of FastAP. The sequencing reaction consisted of 0.5 µl of BigDye Terminator v3.1 and 0.5 µl BigDye Terminator sequencing buffer master mix with 0.64 µM of either the forward or reverse primer and approximately 30 ng purified DNA in a 5 μl reaction. Samples were run on a Techne TC-412 thermocycler at 95°C for 1 minute, 30 cycles of 95°C for 30 seconds, 50°C for 15 seconds, and 60°C for 3 minutes, then 60°C for 5 minutes. Samples were then ethanol precipitated and resuspended in 10 μl of DNAse/RNAse-free H2O (Gibco). Sequencing was executed on an ABI 3730 DNA Analyzer. Sequences were assembled using Geneious v8.0.4.
2. Genome walking was performed to expand on the sequence of the two AMH (Y= Male, X = Autosomal) generally following a modified protocol based on Siebert et al. (1995) and using the primers from Rebrikov et al (2004). Two male and two female DNA templates were used for each amplification. A restriction fragment digest was performed using BbsI, RsaI, NbeI and EcoRI (NEB) with approximately 1 μg DNA from one male and one female lingcod, followed by a phenol:chloroform extraction and ethanol precipitation. Next, NEBNext end repair module protocol was utilized to create blunt ends for all products cut, except those with RsaI. 4.5 μl of fragmented DNA, 1X NEBNext End Repair Reaction Buffer, 5 μl NEBNext End Repair Enzyme Mix, was made up to 50 μl with DNAse/RNAse-free H2O (Gibco). Samples were incubated for 30 minutes at 30°C. A second phenol:chloroform extraction and ethanol precipitation was performed. Next, adaptor AD1 (5'-CTAATACGACTCACTATAGGGCTCGAGCGGCCGCCCGGGCAGGT-3'; 3'-GGCCCGTCCA-5') were ligated using 1 μl T4 DNA ligase (NEB) in 1X ligation buffer (NEB) at 16 °C overnight. 1 μl of EDTA was added and samples were incubated at 72°C for 10 min to terminate reaction. Sample was diluted in 40 μl DNAse/RNAse-free H2O (Gibco). 1 μl samples were used for PCR with primer P1 and GW-X F1, GW-X F2, GW-X R1, GW-X R2, GW-Y F1, GW-Y F2, GW-Y R1, or GW-Y R2 (Additional file 1). The 30 μL PCR mixture contained 1 ul sample, 1X GoTaq Flexi Buffer, 2.5 mM MgCl2, 320 μM of each dNTP (Fisher Scientific), 0.5 μM of forward primer and 0.5 μM reverse primers, 1.5 units GoTaq DNA Polymerase and 1 μl template (Promega reagents). The first reaction was cycled at 95°C for 3 minute, 72°C for 10 minutes, 21 cycles of 95°C for 30 seconds, 62°C for 30 seconds (losing half a degree each cycle) and 72°C for 3 minutes, 19 cycles of 95°C for 30 seconds, 52°C for 30 seconds and 72°C for 3 minutes, then 72°C for 10 minutes. Template diluted 1/200 to be used in second PCR and the next internal primer was used, ie NP1 and GW-X F2, GW-X F3, GW-X R2, GW-X R3, GW-Y F2, GW-Y F3, GW-Y R2, or GW-Y R3 (See list below); otherwise, the same PCR mix as above was used. Second reaction was TDN52LNE 95°C for 3 minutes, 21 cycles of 95°C for 30 seconds, 62°C for 30 seconds (losing half a degree each cycle) and 72°C for 3 minutes, 19 cycles of 95°C for 30 seconds, 52°C for 30 seconds, 72°C for 3 minutes, then 72 for 10 minutes. Clean up performed as per Exo I and FastAP in step 1. Sequencing reaction was performed with as in step 1. Sequencing was executed on an ABI 3730 DNA Analyzer. Sequences were aligned and assembled using Geneious v8.0.4.
3. A second round of genome walking was performed with additional primers. The first round of PCR was P1 with GW-X R4, GW-X R5, GW-Y F1, GW-Y F2, GW-Y R1, or GW-Y R2. The second round of PCR was NP1 and GW-X R5, GW-X R6, GW-Y F2, GW-Y F3, GW-Y R2, or GW-Y R3 (see list below); otherwise, the same protocol was followed.
4. A third round was performed with additional primers. The first round of PCR was GW-X R7, GW-X R8, GW-Y R7 or GW-Y R8. The second round of PCR was NP1 and GW-X R8, GW-X R9, GW-Y R8 or GW-Y R9 (see list below); otherwise, the same protocol was followed.

References:

Siebert PD, Chenchik A, Kellogg DE, Lukyanov KA, Lukyanov SA: **An improved PCR method for walking in uncloned genomic DNA.** *Nucleic Acids Res* 1995, **23**(6)**:**1087-1088.

Rebrikov DV, Desai SM, Siebert PD, Lukyanov SA: **Suppression subtractive hybridization.** In *Methods in Molecular Biology Vol. 258, Gene expression profiling: methods and protocols*. Edited by Shimkets RA. Totowa, NJ: Humana press; 2004:107-134.

Primers for Figure 1 (All amplified following protocol in step 1)

A+B = AMH_F3 = 5’-CTGCTGAAGGCCCTGCAGACG-3’

AMH_R1 = 5’-TACGCCACCGGCACGCAGCA-3’

C = Oelo-AMH-GW-Y-1F = 5’-GAAGGCCCTGCAGACGGTAG-3’

Oelo-AMH-Y-all-4R = 5’-AACCTGCAGCGCGTCGTAGT-3’

D = Oelo-AMH-GW-X-1F = 5’-GAAGGCCCTGCAGACGGTGT-3’

Oelo-AMH-A-all-4R = 5’-CACCTCCAGCGCTTCGTAGG-3’

Primers for Genome Walking (“Y” in the primer name refers to male specific, “X” refers to the shared copy)
Oelo-AMH-GW-X-F1: 5’-CCAGGCCCTGCAGACGGTGT-3’
Oelo-AMH-GW-X-F2: 5’-CCAGAGCTGCAAACAAC-3’
Oelo-AMH-GW-X-F3: 5’-CAACTGGCACGGCTCTTGTT-3’
Oelo-AMH-GW-X-R1: 5’-CGGGGAAAGCACAAGAGCCG-3’
Oelo-AMH-GW-X-R2: 5’-GCCCTCGCTGGGTTGTTTGG-3’
Oelo-AMH-GW-X-R3: 5’-GCACCTCGTAGGCTCGGGAC-3’
Oelo-AMH-GW-Y-F1: 5’-GAAGGCCCTGCAGACGGTAG-3’
Oelo-AMH-GW-Y-F2: 5’-CCAGAGCTGGTTAAACAGC-3’
Oelo-AMH-GW-Y-F3: 5’-CAACTGCCAAGGCTGTTGTT-3’
Oelo-AMH-GW-Y-F4: 5’-GTTGGTAGGCACATGTATCA-3’
Oelo-AMH-GW-Y-F5: 5’-ATGGTACCATGCTCTAAACC-3’
Oelo-AMH-GW-Y-F6: 5’-TTATGGTTGTCCTCAAAGGG-3’
Oelo-AMH-GW-Y-R1: 5’-CAGGGAAAGAACAAGAGCCT-3’
Oelo-AMH-GW-Y-R2: 5’-ACCCTCGCTGGGCTGTTTGA-3’
Oelo-AMH-GW-Y-R3: 5’-GCACCTCGTAGGCACGGGCT-3’
Oelo-AMH-GW-Y-R4: 5’-TATTGTGATTTCCGAGCAGG-3’
Oelo-AMH-GW-Y-R5: 5’-GATCTTCTGCAGGGTGTATG-3’
Oelo-AMH-GW-Y-R6: 5’-TGTGTGGAAAGACTGTTAAGT-3’

Oelo-AMH-GW-X-R4: 5’-TTGGGATTCCCGAGCAGGGGC-3’
Oelo-AMH-GW-X-R5: 5’-TGTGTGGATGGACTGTGAAGT-3’
Oelo-AMH-GW-X-R6: 5’-TGAGATAGAAGGATAAAAGT-3’

Oelo-AMH-GW-X-7R: 5’-ATGAACACACTCGGACCATGAC-3’

Oelo-AMH-GW-X-8R: 5’-TAAGGAGCATTTGAATTTGA-3’

Oelo-AMH-GW-X-9R: 5’-GCTGTGGGTCTGACAAAGATG-3’

Oelo-AMH-GW-Y-7R: 5’-ATGAACACACTTGGACCATGAT-3’

Oelo-AMH-GW-Y-8R: 5’-TAAGGAGCATTTGAGTTATTCT-3’

Oelo-AMH-GW-Y-9R: 5’-ACTGTGGGTCTGACAAAAATA-3’
P1: 5'-CTAATACGACTCACTATAGGG-3'
NP1: 5'-TCGAGCCGGCGCCCGCGCAGG-3'
