# Supplementary material for: A PCR assay detects a male-specific duplicated copy of Anti-Müllerian hormone (amh) in the lingcod (Ophiodon elongatus)
Source: BMC Res Notes. 2016 Apr 22;9:230. doi: 10.1186/s13104-016-2030-6 (PMC4840878; doi:10.1186/s13104-016-2030-6)
Supplement: Supplementary file 3 — 10.1186/s13104-016-2030-6 Partial amh gene sequences. Sequences of male-specific (KP686073) and shared (KP686074) partial amh gene sequences and corresponding alignment (Geneious v8.1.7 default alignment settings). [file 13104_2016_2030_MOESM3_ESM.docx]

>KP686073.2:_Male-Specific

GAGGGCAACGTTCACCAGAAATGGAGGATTTCTGTTGAGATGAAATCCCCCGATATGAGTAAGATATGATGATATGATAGGATTTCTTGCTTCATGAGTACCCTCCTCACATTTTCCTCTCTTTGCAGGCCAAAGCTTAAAAGACATCCTTATTGGTGGAAAATCAAGTAACATCGGCTTGGTTCCGCTTCTGTTTTTCTCTGGGGAAAGAGAAACTGATACAAGGTTGTAATGGCTTTTATGTTCGCTATTAAAATGTTACCAGACCCTTGTCAGAGCGGTGCTCAGCAACTTTTTTTTATATTCTCTTTCTCATATATCATCATAGTTCATCCCTGGCCTTTTCAAAGACCTCCTTCTTCTGCGAGCTGAAGCAGTTCCTGGGTGACGTCCTGCCTCAGGACTACCGTGAGTCCTCTCCACTCCTGCGGGACTCCTTGCAGACCCTGCCTCCCCTGACGTTGGGTTTATCCTCCAGCGACGTCTTGCTGGCGGGACTGATCAACTCCTCCTCCATCACAATCTTCTCCTTCAGCGGCTGGAGCTCCGAGTTTCAGGTGCACCGCAGAGAATTGGCCTTTTCTCCTGCGCTGCTGGAGGAGCTTAAGCGGAGGTTGGAGCAGACCATAGGGAAGATAACAAAGGTCATACGGGAGGAACAAGTGGGTCAGAGGGCCACGGAGAGGCTGAGGAGGCTCAAAGACCTCAGTGTGTTTCCGTTGAATGAACCGGCAGCAGGTGATGTGTACAAGAAAACAGTCTCTTAAAGCAGTTGTTCCCAAATATTTTTGTCAGACCCACAGTGCTGTCAGAATAACTCAAATGCTCCTTAGTCCACATCCCCACATCATGGTCCAAGTGTGTTCATTTGAACTGATTTAAAGTGGATTTTATTGAACCGTTAATGATATAAGAAAGTGTATATAAATGGATGTTACAACATTTTATCATGCAATTAGTCTGTTTAGATCGGTTTAAGTTTGCATTCACACTTCTACCACTTAGTAGCAGAGGGTAGATGCTTCAACCATTCATCAATTACTATAAGCTAATTATTGAAATTGTTTATCCACTACTTGAAGTTATTTCAATCATGTATCCCATTGCTCTAAACCAGGAGGTCATCTCTGAAAGTAGAAAGGATGGACAAGCTGATCTTGGTCTCACTTTAGGGACCCCCAATATAAGACTTCAATGCTCAATGGCCAATAGACATGTGGTGGGAATCAGAATAAAGGTCTAGAAAGCCAACATTTGAAAGGGAAAGAACAAATGAACAAGCAACCAGTGACTTGCTGGTTTGATTTTTGAGGGATCTGTGAAGATCATGACAACAGCTGCATTACGGGGTTTTAGGAATCGAGGATGGACATGATAACAGACAACACTTAAGATTAAAATTCTGTAAAGTATCAGATAAAACAAACTACAACAAATTTGGTTCATTTACTGTATAAAAGCTGTTATTCTTCTATGTCACATTTTCATAATGAAGATAAGTTGATCTACTTAACAGTCTTTCCACACATACACCCTGCAGAAGATCCCCCTGCTCGGAAATCACAATATAATCATGTATTTGCATTATAAATTAGCCCATCTGACATAAATTTGTATTTTTTTAATATCAAGATCCTGCATTACTTTTTAACTGGTGCTTCTGTTACCTTTAGAACAGGGGAGAGCCAGTACCGTGCATTTCTTCTGCTGAAGGCCCTGCAGACGGTAGCCCGTGCCTACGAGGTGCAGAGAGGGTTGCGGGCCACCAGAGCTGGTTCAAACAGCCCAGCGAGGGTCAACATATGTGGGCTGAGGAGCCTCACCGTGTCCCTTGGAAACCTCGGGCTTCCAAAAACCGTCAACATCAACAACTGCCAAGGCTCTTGTTCTTTCCCTGTGACCAACGCCAACAACCATGCCGTCCTGCTCAACTTACACATCGAGAGTGAGAATGTGGACGAGCGGGCGCCATGCTGTGTGCCCGTGGACTACGACGCGCTGCAGGTTGTGGAATTGAAAGGACAAACGACCTACCTCTCCGTGAAACCAGATGTGGTGGCCAAGGAGTGTGGATGCCGCTAAAACTAAATAAATGCTGCTGTAGCGAAAAACAATTCCTAATAGCGCCTCCTACAAATCTTCAAAAGAGCAGCCCCAGCACTACATTACCCCTACATATATAAAAGTTGGTAGGCACATGTATCAAATGGAGACTTAAAAGAAAGTCTCATGGTACCATGCTCTAAACCAGGGATGTCAAACTCATTTTCAACGTGGGCCACATCAGCATTATGGTTGTCCTCAAAGGGCCGGTTGTAACTGTAAAAATTTATAAATGTAACTACTCCCTAACATATTGTTAAATGACTGTCTCTGCATTTAATTATTGTTTATTCAAGTGTAAAAATATAGTACATACATCTATATAGATGTAAAAACGTAT

>KP686074.2:_Shared_Copy

GAGGGCAACGTTCACCAGAAATGGAGGATTTCTGTTGCGACGAAATCCCCCGATATGAGTAAGATACGATGATATGATAGGATTTCTTGTTTCATGAGCACCCTCCTCACATTTTCCTCTCTCTGCAGGCCAAAGCCTAAAAGACATCCTTATTGGTGGAAAATCAAGTAACATCAGCATGGTTCCGCTTCTGCTTTTCTCTGTGGAGAGAGAAACTGATACAAGGTTGTAATGGATTTTATTTTTCCTATTAAAATGTTACCAGACTCTTGTCAGAGCGGGGGTCAGCAACTTTTTTTTTTTATATTCTCTTTCTCAGATATCATCATAGTTCATCCCTGGCCTCTTCACAGACCTCCTTCTTCTGCGAGCTGAAGCGGTTCCTGGGTGACGTCCTGCCTCAGGACCACCGTGAGTCCTCTCCGCTCCTGTGGGACTCCTTGCAGACCCTGCCTCCCCTGACGCTGGGCTTATCCTCCAGCGACGCCCTGCTGGCGGGACTGATCAACTCCTCCTCCATCACCATCTTCTCCTTCAGCAGCTGGAGCTCCGAGTTTCAGGAGCACCGCGGAGAATTGGCCTTTTCTCCTGCGCTGCTGGAGGAGCTCAAGCTGAGGTTGGAGCAGACCTTGGGGAAGATAACGGAGGTCATACGGGAGGAGCAAGTGGGTCAGAGGGCCACGGAGAGGCTGGGGAGGCTCAAGGACCTCAGTGCGTTTCCGTTGAATGAACCGGCAGCAGGTGATGTGTACAAGAAAACAGTCTCTTAAAGCAGTTGTTCCCAAACATCTTTGTCAGACCCACAGCGCTGTCTCAAATTCAAATGCTCCTTAGTCCACATCCCCACGTCATGGTCCGAGTGTGTTCATTTGAACTGATTTAAAGTGGATTTTATTGAACCGTTAATGATATCAAAAAGTGTATATAAGTGGATGTTACAACTTTTTATCATGCAATTAGTCGGTTTAAGTTTGCATTCACAGTTCTACCACTTAAAGTAGCAGAAGGTAGATGCTTCAACCATTCATTCATTACTTTAAGCTAATTATTGAAATTGTTTATCCACTACTTGAGGTTATTTCAATCATGAATCCCATTGCTCTAAACCAGGAGGTCATCTCTGAAAGTAGAGAGGATGGACAAGCTGATCTTGGTCTCACTTTAGGGACCCCCAATCCAAGACTTCAATGCTGAATGGCCAATAGATCTGTGGTGGGAATCAGCACAAAGGTCTAGAAAGCCAACATTTGAAAGGGAAAGAACAAATGAACAAGCAACCAGTGACTTGCTGGTTTGATATTTGAGGGATCTGTAAAGATCACGACAACAGCTGCATTACGGGGTTTTTAGGAATCGAGGATGGACATGATAACAGACAACACTTAAGATTAAAATTCTGTAAAGTATCAGATAAAACACAAACTACAACAAATATTGTTAATTTACTGTATAAAAACTTTTATCCTTCTATCTCACATTTTCCTTATCAAGATAAGTTGAGCTACTTCACAGTCCATCCACACATACACCCTGCAGAAGATGCCCCTGCTCGGGAATCCCAATATAATCACATATTTGCATTATAAATTAGCCAATTTGACATAAATTTGTATTTTTTTTTTATACCAAGATCCTGCATTACTTTTTAACTGGTGCTTCTGTTACCTCTAGAACAGGGGAGAGCCAGTACCGTGCATTTCTTCTGCTGAAGGCCCTGCAGACGGTGTCCCGAGCCTACGAGGTGCAGAGAGGGCTGCGGGCCACCAGAGCTGGTCCAAACAACCCAGCGAGGGCCAACATATGTGGGCTGAGGAGCCTCACCGTGTCCCTGGGAAACCTCGGGCTTCCAAAAACCGTCAACATCAACAACTGCCACGGCTCTTGTGCTTTCCCCGTGACCAACGCCAACAACCACGCCGTCCTGCTCAACTTCCACATCGAGAGTGAGAATGTGGACGAGCGGGCGCCATGCTGTGTGCCCGTGGCCTACGAAGCGCTGGAGGTGGTGGAATTGAAAGAACAAAGGACCTACCTCTCCATGAAACCAGATGTGGTGGCCAAGGAGTGTGGATGCCGCTAAAACTAACGGCTTTCTTCTTGGAGTGKAATTAAGTGATATTTTAAATATTACACATAAACTTGTCAGTGTCATGCAATTGTTACTGCCATTTATTTATTCACACAGTATACAATGGTAGTACATACACGTGTTAATTCACAGAGGTCTCTTTAGGCCATATACTTACATATATGTAGACTATGTATTATTTTCGGTATTCCCACACTTAGAAATGTTCAAGTTTATTCAAACCAAAYAAGTCAAATATAAGTATTATCACCCATCTWTGTCATATTTYTTTTTTTTACTAATCTTATTTGAATAAAAAGCCATTGTTTTATCAGGGCAAAGTTCTTATCATCCATAAGACCGCTTTTTGTGCTTTCATTAAGATTCCATCATATAATTATTATTCAAAAATTGTTAATCACTTAGTGACATTTGAATATAAGATTATGTTACAATTTGGCGGTAAGTGACATTTCTAAATAAGTGATACTAAAA

>Nucleotide alignment 3 Alignment of 2 sequences: KP686073: Male-Specific, KP68
6074: Shared Copy


Score = 9073.5, Identities = 2137/2594 (82%), 
Positives = 2138/2594 (82%), Gaps = 182/2594 (7%)

KP686073: Male-Specific      1 GAGGGCAACGTTCACCAGAAATGGAGGATTTCTGTTGAGATGAAATCCCCCGATATGAGT   60 
                               GAGGGCAACGTTCACCAGAAATGGAGGATTTCTGTTG GA GAAATCCCCCGATATGAGT      
KP686074: Shared Copy        1 GAGGGCAACGTTCACCAGAAATGGAGGATTTCTGTTGCGACGAAATCCCCCGATATGAGT   60 

KP686073: Male-Specific     61 AAGATATGATGATATGATAGGATTTCTTGCTTCATGAGTACCCTCCTCACATTTTCCTCT  120 
                               AAGATA GATGATATGATAGGATTTCTTG TTCATGAG ACCCTCCTCACATTTTCCTCT      
KP686074: Shared Copy       61 AAGATACGATGATATGATAGGATTTCTTGTTTCATGAGCACCCTCCTCACATTTTCCTCT  120 

KP686073: Male-Specific    121 CTTTGCAGGCCAAAGCTTAAAAGACATCCTTATTGGTGGAAAATCAAGTAACATCGGCTT  180 
                               CT TGCAGGCCAAAGC TAAAAGACATCCTTATTGGTGGAAAATCAAGTAACATC GC T      
KP686074: Shared Copy      121 CTCTGCAGGCCAAAGCCTAAAAGACATCCTTATTGGTGGAAAATCAAGTAACATCAGCAT  180 

KP686073: Male-Specific    181 GGTTCCGCTTCTGTTTTTCTCTGGGGAAAGAGAAACTGATACAAGGTTGTAATGGCTTTT  240 
                               GGTTCCGCTTCTG TTTTCTCTG GGA AGAGAAACTGATACAAGGTTGTAATGG TTTT      
KP686074: Shared Copy      181 GGTTCCGCTTCTGCTTTTCTCTGTGGAGAGAGAAACTGATACAAGGTTGTAATGGATTTT  240 

KP686073: Male-Specific    241 ATGTTCGCTATTAAAATGTTACCAGACCCTTGTCAGAGCGGTGCTCAGCAACTT---TTT  297 
                               AT TT  CTATTAAAATGTTACCAGAC CTTGTCAGAGCGG G TCAGCAACTT   TTT      
KP686074: Shared Copy      241 ATTTTTCCTATTAAAATGTTACCAGACTCTTGTCAGAGCGGGGGTCAGCAACTTTTTTTT  300 

KP686073: Male-Specific    298 TTTATATTCTCTTTCTCATATATCATCATAGTTCATCCCTGGCCTTTTCAAAGACCTCCT  357 
                               TTTATATTCTCTTTCTCA ATATCATCATAGTTCATCCCTGGCCT TTCA AGACCTCCT      
KP686074: Shared Copy      301 TTTATATTCTCTTTCTCAGATATCATCATAGTTCATCCCTGGCCTCTTCACAGACCTCCT  360 

KP686073: Male-Specific    358 TCTTCTGCGAGCTGAAGCAGTTCCTGGGTGACGTCCTGCCTCAGGACTACCGTGAGTCCT  417 
                               TCTTCTGCGAGCTGAAGC GTTCCTGGGTGACGTCCTGCCTCAGGAC ACCGTGAGTCCT      
KP686074: Shared Copy      361 TCTTCTGCGAGCTGAAGCGGTTCCTGGGTGACGTCCTGCCTCAGGACCACCGTGAGTCCT  420 

KP686073: Male-Specific    418 CTCCACTCCTGCGGGACTCCTTGCAGACCCTGCCTCCCCTGACGTTGGGTTTATCCTCCA  477 
                               CTCC CTCCTG GGGACTCCTTGCAGACCCTGCCTCCCCTGACG TGGG TTATCCTCCA      
KP686074: Shared Copy      421 CTCCGCTCCTGTGGGACTCCTTGCAGACCCTGCCTCCCCTGACGCTGGGCTTATCCTCCA  480 

KP686073: Male-Specific    478 GCGACGTCTTGCTGGCGGGACTGATCAACTCCTCCTCCATCACAATCTTCTCCTTCAGCG  537 
                               GCGACG C TGCTGGCGGGACTGATCAACTCCTCCTCCATCAC ATCTTCTCCTTCAGC       
KP686074: Shared Copy      481 GCGACGCCCTGCTGGCGGGACTGATCAACTCCTCCTCCATCACCATCTTCTCCTTCAGCA  540 

KP686073: Male-Specific    538 GCTGGAGCTCCGAGTTTCAGGTGCACCGCAGAGAATTGGCCTTTTCTCCTGCGCTGCTGG  597 
                               GCTGGAGCTCCGAGTTTCAGG GCACCGC GAGAATTGGCCTTTTCTCCTGCGCTGCTGG      
KP686074: Shared Copy      541 GCTGGAGCTCCGAGTTTCAGGAGCACCGCGGAGAATTGGCCTTTTCTCCTGCGCTGCTGG  600 

KP686073: Male-Specific    598 AGGAGCTTAAGCGGAGGTTGGAGCAGACCATAGGGAAGATAACAAAGGTCATACGGGAGG  657 
                               AGGAGCT AAGC GAGGTTGGAGCAGACC T GGGAAGATAAC  AGGTCATACGGGAGG      
KP686074: Shared Copy      601 AGGAGCTCAAGCTGAGGTTGGAGCAGACCTTGGGGAAGATAACGGAGGTCATACGGGAGG  660 

KP686073: Male-Specific    658 AACAAGTGGGTCAGAGGGCCACGGAGAGGCTGAGGAGGCTCAAAGACCTCAGTGTGTTTC  717 
                               A CAAGTGGGTCAGAGGGCCACGGAGAGGCTG GGAGGCTCAA GACCTCAGTG GTTTC      
KP686074: Shared Copy      661 AGCAAGTGGGTCAGAGGGCCACGGAGAGGCTGGGGAGGCTCAAGGACCTCAGTGCGTTTC  720 

KP686073: Male-Specific    718 CGTTGAATGAACCGGCAGCAGGTGATGTGTACAAGAAAACAGTCTCTTAAAGCAGTTGTT  777 
                               CGTTGAATGAACCGGCAGCAGGTGATGTGTACAAGAAAACAGTCTCTTAAAGCAGTTGTT      
KP686074: Shared Copy      721 CGTTGAATGAACCGGCAGCAGGTGATGTGTACAAGAAAACAGTCTCTTAAAGCAGTTGTT  780 

KP686073: Male-Specific    778 CCCAAATATTTTTGTCAGACCCACAGTGCTGTCAGAATAACTCAAATGCTCCTTAGTCCA  837 
                               CCCAAA AT TTTGTCAGACCCACAG GCTGTC  A  AA TCAAATGCTCCTTAGTCCA      
KP686074: Shared Copy      781 CCCAAACATCTTTGTCAGACCCACAGCGCTGTCTCA--AATTCAAATGCTCCTTAGTCCA  838 

KP686073: Male-Specific    838 CATCCCCACATCATGGTCCAAGTGTGTTCATTTGAACTGATTTAAAGTGGATTTTATTGA  897 
                               CATCCCCAC TCATGGTCC AGTGTGTTCATTTGAACTGATTTAAAGTGGATTTTATTGA      
KP686074: Shared Copy      839 CATCCCCACGTCATGGTCCGAGTGTGTTCATTTGAACTGATTTAAAGTGGATTTTATTGA  898 

KP686073: Male-Specific    898 ACCGTTAATGATATAAGAAAGTGTATATAAATGGATGTTACAACATTTTATCATGCAATT  957 
                               ACCGTTAATGATAT A AAAGTGTATATAA TGGATGTTACAAC TTTTATCATGCAATT      
KP686074: Shared Copy      899 ACCGTTAATGATATCAAAAAGTGTATATAAGTGGATGTTACAACTTTTTATCATGCAATT  958 

KP686073: Male-Specific    958 AGTCTGTTTAGATCGGTTTAAGTTTGCATTCACACTTCTACCACTTA--GTAGCAGAGGG 1015 
                               AG          TCGGTTTAAGTTTGCATTCACA TTCTACCACTTA  GTAGCAGA GG      
KP686074: Shared Copy      959 AG----------TCGGTTTAAGTTTGCATTCACAGTTCTACCACTTAAAGTAGCAGAAGG 1008 

KP686073: Male-Specific   1016 TAGATGCTTCAACCATTCATCAATTACTATAAGCTAATTATTGAAATTGTTTATCCACTA 1075 
                               TAGATGCTTCAACCATTCAT  ATTACT TAAGCTAATTATTGAAATTGTTTATCCACTA      
KP686074: Shared Copy     1009 TAGATGCTTCAACCATTCATTCATTACTTTAAGCTAATTATTGAAATTGTTTATCCACTA 1068 

KP686073: Male-Specific   1076 CTTGAAGTTATTTCAATCATGTATCCCATTGCTCTAAACCAGGAGGTCATCTCTGAAAGT 1135 
                               CTTGA GTTATTTCAATCATG ATCCCATTGCTCTAAACCAGGAGGTCATCTCTGAAAGT      
KP686074: Shared Copy     1069 CTTGAGGTTATTTCAATCATGAATCCCATTGCTCTAAACCAGGAGGTCATCTCTGAAAGT 1128 

KP686073: Male-Specific   1136 AGAAAGGATGGACAAGCTGATCTTGGTCTCACTTTAGGGACCCCCAATATAAGACTTCAA 1195 
                               AGA AGGATGGACAAGCTGATCTTGGTCTCACTTTAGGGACCCCCAAT  AAGACTTCAA      
KP686074: Shared Copy     1129 AGAGAGGATGGACAAGCTGATCTTGGTCTCACTTTAGGGACCCCCAATCCAAGACTTCAA 1188 

KP686073: Male-Specific   1196 TGCTCAATGGCCAATAGACATGTGGTGGGAATCAGAATAAAGGTCTAGAAAGCCAACATT 1255 
                               TGCT AATGGCCAATAGA  TGTGGTGGGAATCAG A AAAGGTCTAGAAAGCCAACATT      
KP686074: Shared Copy     1189 TGCTGAATGGCCAATAGATCTGTGGTGGGAATCAGCACAAAGGTCTAGAAAGCCAACATT 1248 

KP686073: Male-Specific   1256 TGAAAGGGAAAGAACAAATGAACAAGCAACCAGTGACTTGCTGGTTTGATTTTTGAGGGA 1315 
                               TGAAAGGGAAAGAACAAATGAACAAGCAACCAGTGACTTGCTGGTTTGAT TTTGAGGGA      
KP686074: Shared Copy     1249 TGAAAGGGAAAGAACAAATGAACAAGCAACCAGTGACTTGCTGGTTTGATATTTGAGGGA 1308 

KP686073: Male-Specific   1316 TCTGTGAAGATCATGACAACAGCTGCATTACGGGGTTT-TAGGAATCGAGGATGGACATG 1374 
                               TCTGT AAGATCA GACAACAGCTGCATTACGGGGTTT TAGGAATCGAGGATGGACATG      
KP686074: Shared Copy     1309 TCTGTAAAGATCACGACAACAGCTGCATTACGGGGTTTTTAGGAATCGAGGATGGACATG 1368 

KP686073: Male-Specific   1375 ATAACAGACAACACTTAAGATTAAAATTCTGTAAAGTATCAGATAAAACA--AACTACAA 1432 
                               ATAACAGACAACACTTAAGATTAAAATTCTGTAAAGTATCAGATAAAACA  AACTACAA      
KP686074: Shared Copy     1369 ATAACAGACAACACTTAAGATTAAAATTCTGTAAAGTATCAGATAAAACACAAACTACAA 1428 

KP686073: Male-Specific   1433 CAAATTTGGTTCATTTACTGTATAAAAGCTGTTATTCTTCTATGTCACATTTTCATAATG 1492 
                               CAAAT T GTT ATTTACTGTATAAAA CT TTAT CTTCTAT TCACATTTTC T AT       
KP686074: Shared Copy     1429 CAAATATTGTTAATTTACTGTATAAAAACTTTTATCCTTCTATCTCACATTTTCCTTATC 1488 

KP686073: Male-Specific   1493 AAGATAAGTTGATCTACTTAACAGTCTTTCCACACATACACCCTGCAGAAGATCCCCCTG 1552 
                               AAGATAAGTTGA CTACTT ACAGTC  TCCACACATACACCCTGCAGAAGAT CCCCTG      
KP686074: Shared Copy     1489 AAGATAAGTTGAGCTACTTCACAGTCCATCCACACATACACCCTGCAGAAGATGCCCCTG 1548 

KP686073: Male-Specific   1553 CTCGGAAATCACAATATAATCATGTATTTGCATTATAAATTAGCCCATCTGACATAAATT 1612 
                               CTCGG AATC CAATATAATCA  TATTTGCATTATAAATTAGCC AT TGACATAAATT      
KP686074: Shared Copy     1549 CTCGGGAATCCCAATATAATCACATATTTGCATTATAAATTAGCCAATTTGACATAAATT 1608 

KP686073: Male-Specific   1613 TGTA--TTTTTTTAATATCAAGATCCTGCATTACTTTTTAACTGGTGCTTCTGTTACCTT 1670 
                               TGTA  TTTTTTT ATA CAAGATCCTGCATTACTTTTTAACTGGTGCTTCTGTTACCT       
KP686074: Shared Copy     1609 TGTATTTTTTTTTTATACCAAGATCCTGCATTACTTTTTAACTGGTGCTTCTGTTACCTC 1668 

KP686073: Male-Specific   1671 TAGAACAGGGGAGAGCCAGTACCGTGCATTTCTTCTGCTGAAGGCCCTGCAGACGGTAGC 1730 
                               TAGAACAGGGGAGAGCCAGTACCGTGCATTTCTTCTGCTGAAGGCCCTGCAGACGGT  C      
KP686074: Shared Copy     1669 TAGAACAGGGGAGAGCCAGTACCGTGCATTTCTTCTGCTGAAGGCCCTGCAGACGGTGTC 1728 

KP686073: Male-Specific   1731 CCGTGCCTACGAGGTGCAGAGAGGGTTGCGGGCCACCAGAGCTGGTTCAAACAGCCCAGC 1790 
                               CCG GCCTACGAGGTGCAGAGAGGG TGCGGGCCACCAGAGCTGGT CAAACA CCCAGC      
KP686074: Shared Copy     1729 CCGAGCCTACGAGGTGCAGAGAGGGCTGCGGGCCACCAGAGCTGGTCCAAACAACCCAGC 1788 

KP686073: Male-Specific   1791 GAGGGTCAACATATGTGGGCTGAGGAGCCTCACCGTGTCCCTTGGAAACCTCGGGCTTCC 1850 
                               GAGGG CAACATATGTGGGCTGAGGAGCCTCACCGTGTCCCT GGAAACCTCGGGCTTCC      
KP686074: Shared Copy     1789 GAGGGCCAACATATGTGGGCTGAGGAGCCTCACCGTGTCCCTGGGAAACCTCGGGCTTCC 1848 

KP686073: Male-Specific   1851 AAAAACCGTCAACATCAACAACTGCCAAGGCTCTTGTTCTTTCCCTGTGACCAACGCCAA 1910 
                               AAAAACCGTCAACATCAACAACTGCCA GGCTCTTGT CTTTCCC GTGACCAACGCCAA      
KP686074: Shared Copy     1849 AAAAACCGTCAACATCAACAACTGCCACGGCTCTTGTGCTTTCCCCGTGACCAACGCCAA 1908 

KP686073: Male-Specific   1911 CAACCATGCCGTCCTGCTCAACTTACACATCGAGAGTGAGAATGTGGACGAGCGGGCGCC 1970 
                               CAACCA GCCGTCCTGCTCAACTT CACATCGAGAGTGAGAATGTGGACGAGCGGGCGCC      
KP686074: Shared Copy     1909 CAACCACGCCGTCCTGCTCAACTTCCACATCGAGAGTGAGAATGTGGACGAGCGGGCGCC 1968 

KP686073: Male-Specific   1971 ATGCTGTGTGCCCGTGGACTACGACGCGCTGCAGGTTGTGGAATTGAAAGGA 2022 
                               ATGCTGTGTGCCCGTGG CTACGA GCGCTG AGGT GTGGAATTGAAAG A      
KP686074: Shared Copy     1969 ATGCTGTGTGCCCGTGGCCTACGAAGCGCTGGAGGTGGTGGAATTGAAAGAA 2020 

KP686073: Male-Specific   2025 CAAACGACCTACCTCTCCGTGAAACCAGATGTGGTGGCCAAGGAGTGTGGATGCCGCTAA 2084 
                               CAAA GACCTACCTCTCC TGAAACCAGATGTGGTGGCCAAGGAGTGTGGATGCCGCTAA      
KP686074: Shared Copy     2984 CAAAGGACCTACCTCTCCATGAAACCAGATGTGGTGGCCAAGGAGTGTGGATGCCGCTAA 3043 

KP686073: Male-Specific   2085 AACTAAATAAATGCTGCT-GTAGCGAAAAACAATTCCTAATAGCGCCTCCTACAAATCTT 2143 
                               AACTAA     T CT CT G AG G  AA  AA T  TA T      T  TACA AT         
KP686074: Shared Copy     3044 AACTAACGGCTTTCTTCTTGGAGTG-KAATTAAGTGATA-TTTTAAATATTACACAT--- 3098 

KP686073: Male-Specific   2144 CAAAAGAG-CAGCCCCA-GCACTACATTACCCCTACATATATAA-AAGTTGGTAGGCACA 2200 
                                AAA   G CAG   CA GCA T   TTAC  C A  TAT TA   A    GTA  CA        
KP686074: Shared Copy     3099 -AAACTTGTCAGTGTCATGCAATT-GTTACTGCCATTTATTTATTCACACAGTATACAAT 3156 

KP686073: Male-Specific   2201 TGTATCAAATGGA-GACTTAAA--AGA-AAGTCTCATGGTACCATGCTCTAAACCAGGGA 2256 
                                GTA  A AT  A G  TTAA   A A A GTCTC T    CCAT   CT A  CA   A      
KP686074: Shared Copy     3157 GGTAGTACATACACGTGTTAATTCACAGAGGTCTCTTTAGGCCATATACTTA--CATATA 3214 

KP686073: Male-Specific   2257 TGTCAAACTCATTTTCAACGTGGGCCACATCAGCATTATGGTTGTCCTCAAAGGGCCGGT 2316 
                               TGT A ACT   T T A   T GG      CA   TTA    TGT  TCAA   G    T      
KP686074: Shared Copy     3215 TGT-AGACTATGTATTATTTTCGGTATTCCCACACTTAGAAATGT--TCAA---GTTTAT 3268 

KP686073: Male-Specific   2317 TGTAACTGTAAAAATTTATAAATGTAACTACTCCCTAACATAT-TGTTAAATGACTGTCT 2375 
                               T  AAC   AAA A  T  AAAT TAA TA T  C   CAT T TGT A AT  +T T T      
KP686074: Shared Copy     3269 TCAAAC--CAAAYAAGT-CAAATATAAGTATTATCACCCATCTWTGTCATATTTYTTTTT 3325 

KP686073: Male-Specific   2376 CTGCATTTAATTATT-GTTTATTCAAGTGTAAAAATAT-AGTACATACATCTATAT-AGA 2432 
                                T   T    TTATT G  TA   A    T     TAT AG  CA A  TCT TAT A        
KP686074: Shared Copy     3326 TTTACTAATCTTATTTGAATAAAAAGCCATTGTTTTATCAGGGCAAAGTTCT-TATCATC 3384 

KP686073: Male-Specific   2433 TGTAAAAACGTAT 2445 
                                 TAA A CG  T      
KP686074: Shared Copy     3385 CATAAGACCGCTT 3397 

KP686073: Male-Specific        ------------------------------------------------------------      
                                                                                                 
KP686074: Shared Copy     3417 TTTGTGCTTTCATTAAGATTCCATCATATAATTATTATTCAAAAATTGTTAATCACTTAG 3476 

KP686073: Male-Specific        ------------------------------------------------------------      
                                                                                                 
KP686074: Shared Copy     3477 TGACATTTGAATATAAGATTATGTTACAATTTGGCGGTAAGTGACATTTCTAAATAAGTG 3536 

KP686073: Male-Specific        ---------      
                                              
KP686074: Shared Copy     3537 ATACTAAAA 3545
