# Supplementary figures and images for: A PCR assay detects a male-specific duplicated copy of Anti-Müllerian hormone (amh) in the lingcod (Ophiodon elongatus)
Source: BMC Res Notes. 2016 Apr 22;9:230. doi: 10.1186/s13104-016-2030-6 (PMC4840878; doi:10.1186/s13104-016-2030-6)

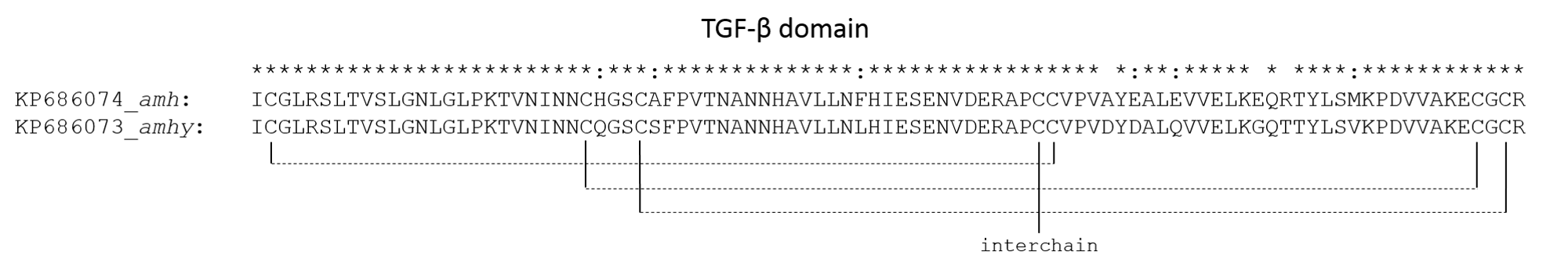

Supplement: Supplementary file 4 — 10.1186/s13104-016-2030-6 TGF-β domain. Translation of TGF-β domain and identification of conserved cysteines between amh and amhy. [file 13104_2016_2030_MOESM4_ESM.png]
